# Reveal—visual eQTL analytics

**DOI:** 10.1093/bioinformatics/bts382

**Published:** 2012-09-03

**Authors:** Günter Jäger, Florian Battke, Kay Nieselt

**Affiliations:** ^1^Integrative Transcriptomics, Center for Bioinformatics, University of Tübingen, 72076 Tübingen, Germany

## Abstract

**Motivation:** The analysis of expression quantitative trait locus (eQTL) data is a challenging scientific endeavor, involving the processing of very large, heterogeneous and complex data. Typical eQTL analyses involve three types of data: sequence-based data reflecting the genotypic variations, gene expression data and meta-data describing the phenotype. Based on these, certain genotypes can be connected with specific phenotypic outcomes to infer causal associations of genetic variation, expression and disease.

To this end, statistical methods are used to find significant associations between single nucleotide polymorphisms (SNPs) or pairs of SNPs and gene expression. A major challenge lies in summarizing the large amount of data as well as statistical results and to generate informative, interactive visualizations.

**Results:** We present Reveal, our visual analytics approach to this challenge. We introduce a graph-based visualization of associations between SNPs and gene expression and a detailed genotype view relating summarized patient cohort genotypes with data from individual patients and statistical analyses.

**Availability:** Reveal is included in Mayday, our framework for visual exploration and analysis. It is available at http://it.inf.uni-tuebingen.de/software/reveal/.

**Contact:** guenter.jaeger@uni-tuebingen.de

## 1 INTRODUCTION

The risk to come down with a complex disease such as cancer or diabetes can be influenced by genetic variations. Genome-wide association studies (GWASs) help with the identification of such risk-increasing local DNA sequence variants. The ultimate goal is to generate a complete view of the variability of a genome of individuals. Modern microarray and sequencing technologies already allow for the compilation of hundreds of thousands of so-called single nucleotide polymorphisms (SNPs). In typical GWAS studies, linkage analyses are performed, where each SNP is tested for association with a specific phenotype of a disease. Thus, one or more SNP genotypes are sought whose frequency can be correlated with a disease. However, for many thus identified SNPs, little is known about the molecular mechanisms underlying their phenotypic manifestation.

Expression quantitative trait loci (eQTL) studies go one step further, involving three types of data: sequence-based data reflecting the genotypic variations, gene expression data and meta-data describing the phenotype, e.g. the severity of disease or speed of progression. This analysis is therefore highly challenging, since it involves the scalable processing of very large, heterogeneous and complex data. For example, the HapMap project (Gibbs *et al.*, 2003) has generated genotype data for *>*3 million human SNPs. Such genotype data contain for each individual and each assessed SNP a pair of nucleotides, reflecting the two alleles (maternal and paternal) of the individual.

Furthermore, the expression values of tens of thousands of genes from thousands of individuals are gathered. Statistical tests are used to compute for each pair of SNP and transcript whether a significant association exists between the presence of polymorphisms and a difference in gene expression. These statistical analyses often generate a large number of SNPs being either directly or indirectly associated with the expression of specific genes.

The goal of eQTL data analysis is to generate a comprehensive picture that connects certain genotypes (individual genetic information) with specific phenotypic outcomes. Based on this aggregated view of the data, analysts (e.g. biologists and bioinformaticians) are enabled to infer causal associations of genetic variation, expression and disease, i.e. to identify the genetic basis for phenotypic variations. More generally, the ultimate goal is to find a network of interacting genes whose expression changes are correlated with genetic variation, allowing for a prognosis of disease based on observed patient phenotypes.

A typical workflow for eQTL data analysis consists of applying either machine-learning methods or statistical tests to extract significant associations. The results, however, are often extremely difficult to interpret. Biologists or clinicians are mainly interested in the identification of correlated genes together with their associated SNPs, and the phenotype connection. Visualizations in this field are highly valuable and require views that can handle interaction, filtering and zooming with these complex data. The most important aspect of visual analytics in this context lies in data aggregation, filtering and the creation of meaningful summaries to allow researchers to extract the few important associations with clinical significance from the enormous amount of input data.

Here, we present Reveal, a visual analytics approach to this challenge. A proof-of-concept implementation of Reveal was submitted to the BioVis 2011 Contest (part of the IEEE VisWeek 2011 BioVis symposium). Every year, this newly inaugurated contest focuses on a specific biological application domain and solicits submissions combining data analysis and visualization. Last year's domain was eQTL analysis and Reveal was chosen as the visualization experts' favorite solution.

We have now significantly expanded our initial implementation. We included a new visualization to analyze distributions of genetic variations in more detail. Furthermore, we integrated Reveal into our visual analytics software Mayday (Battke *et al.*, 2010), allowing for combined and highly interactive analyses of genotypic and expression data as well as meta-data (e.g. disease phenotype). We apply Reveal to the BioVis 2011 Contest dataset and discuss results generated.

## 2 RELATED WORK

A traditional visualization for genotype–phenotype associations are Manhattan plots. They are a special kind of scatter plot of significance, represented as the negative logarithm of the *P*-value, of a trait against chromosomal location. All SNPs lying above a pre-chosen significance threshold can easily be detected visually if a conservative threshold is chosen. A popular stand-alone tool is WGAViewer (Ge *et al.*, 2008) which offers an interactive Manhattan plot embedded into an annotation environment in order to help identify those associations with large biological relevance.

Genevar (Yang *et al.*, 2010) combines a database and a visualization of SNPs associated with gene expression using Manhattan plots. Although the Manhattan plot is useful for a small number of traits, problems arise with a fully genome-wide screen where millions of SNPs, for example in the human genome, are tested for association with hundreds or thousands of traits.

Many applications in this area combine the (visual) detection of significant SNPs with the genomic context, mainly integrated in genome browsers. Two examples for this approach are eQTL Explorer (Mueller *et al.*, 2006) and the AssociationViewer (Martin *et al.*, 2009). Genome browsers have the advantage of allowing simultaneous display of data and annotation in separate tracks. This is useful if results of several studies shall be integrated into one view. Furthermore, genome browsers are highly scalable and interactive. However, if the eQTL data are collected in a genome-wide screen, the genome browsing approach is of limited usability. In particular, it will not allow for an identification of a comprehensive pattern of association.

eQTL Viewer (Zou *et al.*, 2007) is a web-based tool that visualizes the relationships between the expression traits and candidate genes in the eQTL regions. It displays eQTL mapping results and allows one to generate plots in SVG format that are interactive and allow the superimposition of mapped data and biological annotations.

A specialized application to visualize all HapMap genotypes together with gene expression levels is SNPexp (Holm *et al.*, 2010). Although it is certainly helpful to offer ready-made solutions for data that are often used, the specialization on one particular dataset also limits the applicability of a tool. Furthermore, as with all web services, users of SNPexp might not be willing to work on the web, especially when sensitive data are concerned. At the time of writing, SNPexp seemed to suffer from a problem with internal data files, limiting our ability to evaluate it in practice.

A recently published study of the genome-wide association of significant SNPs with celiac disease showed that a large number of genes from regions within 500 kb of SNPs associated with case/control differences are highly co-expressed (Dubois *et al.*, 2010). This impressive study is an example demonstrating that for eQTL studies where SNPs are analyzed in the context of many genes, networks that visualize both the interaction of genes as well as the relationship of each gene with multiple SNPs are needed.

A visual analytics approach is followed with GenAMap (Curtis *et al.*, 2011). GenAMap's strategy implements a typical association mapping analysis workflow. Starting from quality-controlled data of SNPs and phenotypes, machine-learning methods are used to identify patterns in the data (e.g. patterns for SNPs and their associated traits) which are then made available to the researchers for interactive exploration using statistical/mathematical programming environments and manual reformatting for the next step of machine learning, where structured association results are produced. These can again be explored manually. This approach is extremely flexible and very promising, yet clearly engineered towards bioinformaticians as opposed to researchers with a background in biology and medicine.

Among the commercial tools for this type of data, the SNP and Variation Suite (SVS 7) by (Golden Helix, 2012) offer various statistical tests and visualization within an integrated genome browser. Agilent's GeneSpring (Agilent, 2012) has a number of statistical and visualization methods for GWASs, however, no specific eQTL analysis methods are offered. Illumina GenomeStudio™ (Illumina, 2012) also offers an integrated use of PLINK as well as a QTL viewer. In addition, several third-party tools for eQTL data analysis are supported within Illumina's workflow.

However, most commercial tools do not yet offer sophisticated and specific eQTL data analysis or visualization methods.

In contrast to the described approaches, Reveal offers a graph-based visualization that indirectly implies a gene regulatory network on the basis of associations of SNPs with specific genes and, in addition to that, allows one to combine the gathered results with a detailed gene expression analysis.

## 3 Reveal

We present Reveal, a tool for visual analyses of eQTL data. It is included in our visual analytics tool Mayday through which further visualizations and methods can be included in the analytical process. Reveal is based on three views, one focusing on the network of associations defined by the influences of SNPs on gene expression, the second providing detailed information on patient cohort genotypes grouped by meta-data and the third showing a traditional heatmap of the gene expression values.

### 3.1 Association gene network

The starting point of an analysis using Reveal is a list of SNPs and genes, and data from a patient cohort covering the presence of the sequence polymorphisms and the expression values of the genes. In addition, patients are assigned to one of two groups depending on their outcome, e.g. into ‘affected’ and ‘unaffected’ patients.

**Fig. 1.**
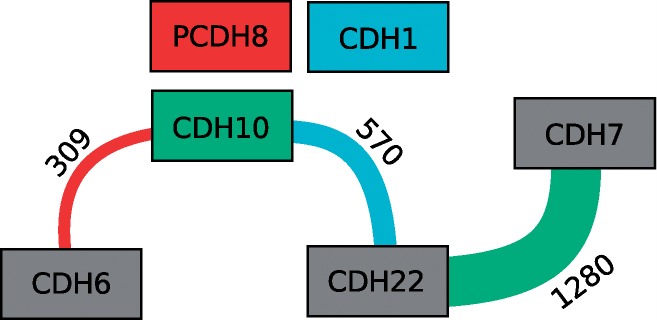
Elements of the association graph. Nodes represent genes, edges represent significant SNP pairs. Genes significantly associated with SNP pairs are colored using a distinct color, genes with no significant association are drawn with gray fill. Each edge conveys four pieces of information: an edge *e* of weight *w* starting in node *s*, ending in node *t* and drawn with color *c* represents *w* SNP pairs. Each of them has one SNP in gene *s* and one in gene *t*, and they are significantly associated with the expression of the gene whose node is filled with color *c*. In this example, 1280 SNP pairs with SNPs in CDH22 and CDH7 are associated with the expression of CDH10, while 570 SNP pairs with SNPs in CDH22 and CDH10 are associated with the expression of CDH1, which does not contain any significant SNPs, itself

On these data, statistical methods such as the PLINK tool (Purcell *et al.*, 2007) can be used to compute the significance of the association between any SNP (or pair of SNPs) and differences in gene expression. These significances are expressed as *P*-values. We use these pairs of SNPs to construct and display an association graph (Fig. 1).

To construct the graph, each gene in the dataset is represented by a node. We use the chromosomal location of SNPs to associate them with genes, i.e. each SNP is associated with its closest gene (lying inside or close to the gene's locus). This allows us, for each gene, to determine the number of significant SNP pairs with at least one pair partner associated with that gene. Nodes of genes with at least one such pair are assigned a unique color, all other nodes are painted using a gray fill.

Edges are added between nodes as follows: based on the *P*-values computed for the association between SNP pairs and gene expression, create a triple < *g_i_*,*g_j_*, *g_k_* > of genes for each SNP pair with partners in *g_i_* and *g_j_* which is significantly associated with the gene expression of *g_k_*. For each *g_k_*, add an edge between the nodes of *g_i_* and *g_j_* with weight *w* = |{< *g_i_*,*g_j_*,*g_k_* >}| and color *c*(*g_k_*). As SNPs located in or close to *g_i_* and *g_j_* can form pairs which influence the expression of different target genes, the graph can contain multi-edges which differ only in color (and probably also in weight).

To increase visual clarity, users can interactively manipulate a minimum edge weight threshold *τ*, such that only edges with *w* ≥ *τ* are displayed. User interaction further includes panning, rotating and zooming, as well as rearranging nodes either manually or using layout algorithms (provided by the Jung library (O'Madadhain *et al.*, 2005)). Expression values can be included in the visualization by mapping them to the node size. Furthermore, if a node is selected, either all neighboring nodes or all edges drawn with the same color as the selected node, i.e. edges containing SNP pairs that significantly influence the expression of the selected node, can be highlighted to improve the visual analytical process. Using the threshold *τ*, users can narrow down the set of SNP pairs they want to investigate further. Another possibility is to select one or more edges and only concentrate on SNPs represented by those edges. Such a filtered set of SNP pairs can then be used to create a genotype view for a more detailed inspection.

### 3.2 Genotype view: SNPs and meta-data

**Fig. 2.**
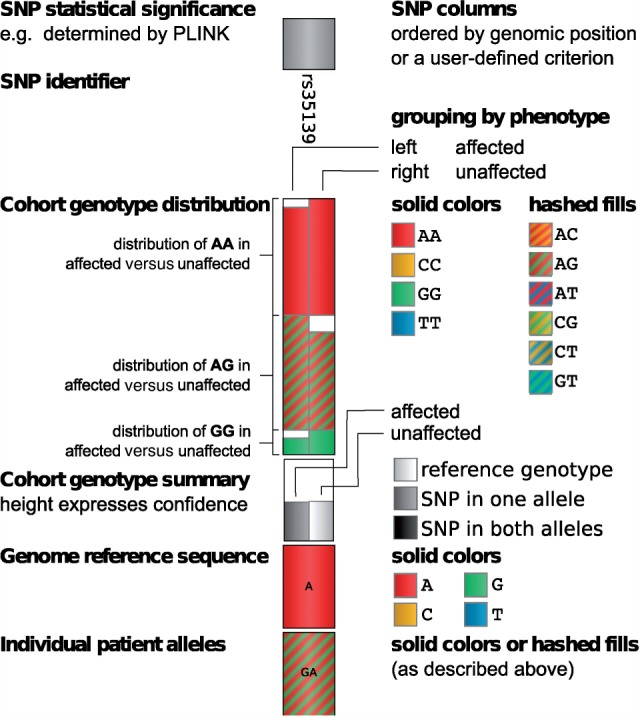
A single SNP column of the genotype view. The column consists of six elements. The ‘SNP statistical significance bar’ displays the −log*P*-value of the association of the SNP with one of the quantitative traits. Depending on the data, the ‘SNP identifier’ is either an #rs number identifying the SNP or the SNP's chromosomal location. Solid and hashed fills are used to encode for the different allelic combinations. The ‘cohort genotype distribution’ shows stacked distributions of allelic combinations in the ‘affected’ (left) and ‘unaffected’ (right) patients. The stacked distributions are ordered alphabetically and distributions of the same allelic combination in the affected and unaffected groups are aligned horizontally for direct comparison. The ‘cohort genotype summary’ is an aggregated view of the cohort genotype distribution where the height of each box expresses confidence in the affected and unaffected groups. The ‘genome reference sequence’ box is colored according to the SNP's reference nucleotide. The ‘individual patient alleles’ box shows an individual's allelic combination encoded with the same solid and hashed fills used for the cohort genotype distribution box

Although the association network nicely visualizes gene-to-gene interactions based on SNP presence, it gives no detailed information about SNPs involved in the network. However, including additional information such as statistical significance values or the distribution of specific SNPs in the patient cohort can be of great benefit to the analytical process, since it provides further insight into a possible connection of genotype and disease state. Therefore, we introduce a second visualization, namely the genotype view, that shows detailed information about each SNP, integrating genotype information for the complete patient cohort as well as for an individual patient (Fig. 2). It is basically a tabular view, where each SNP is represented by one column. Columns are initially ordered according to the genomic position of the visualized SNPs. Other orderings are also possible, e.g. according to the majority genotype in the patient cohort.

The view itself is subdivided into five sectors: in the bottom row of the table, an individual patient's genotype is displayed using color to represent the different nucleotide combinations (solid colors for homozygous genotypes and hashed fills for heterozygous genotypes). The displayed individual can be selected by the user. Above it, the reference genotype (based on the human genome reference assembly) is shown for comparison. Further up, the patient cohort is presented in aggregated form: based on the meta-data, the cohort is split into groups, e.g. ‘affected’ versus ‘unaffected’ and aggregated independently for each group. Here, we use the same aggregation strategy as iHat (Heinrich *et al.*, 2012), our previously published prototype tool for visual analytics of GWASs.

Firstly, a group summary is computed (similar to the ‘consensus’ in multiple alignments) to reflect the majority of genotypes, simplified to one of three states and mapped to gray-scale values: identical to the reference (white), polymorphism in one allele and reference in the other (gray), polymorphism in both alleles (black). Boxes for the two groups are displayed next to each other by default. The strength of the consensus, i.e. the percentage of patients with the majority phenotype, is mapped to the height of the box such that the box has maximum height if all patients in the group show the same (simplified) genotype. Users can switch to a stacked view interactively, displaying the boxes on top of each other rather than next to each other. This enables to quickly spot interesting columns especially when a large number of columns are shown (zoomed-out overview; Fig. 5). By zooming in, users can focus on the more detailed cohort distribution view offered above the cohort aggregate row.

In the full cohort distribution row shown above the cohort genome summary plot, each SNP column is divided into two sub-columns to display the differences between the patients in the ‘affected’ and ‘unaffected’ group. The distribution of real genotypes in the patient cohort groups is visualized using a stacked bar plot and the same coloring as for the individual patient alleles. The focus of this visualization is to allow users to compare the genotypes between the cohort groups (e.g. ‘affected’ versus ‘unaffected’) with respect to the respective SNP position. To facilitate this, bars for identical genotypes (e.g. AC, shown with red/yellow hashed fill) are aligned horizontally to be directly comparable.

At the top of each SNP column, the SNP identifier (or genomic location, depending on the input data) is displayed together with a bar plot showing the negative logarithm of the *P*-value assigned to this SNP position. The bar plot uses a similar approach to display SNP significance values as the traditional Manhattan plot.

Interactive tooltips provide additional information about each of the five sectors in a SNP column, as for example the values from the genotype distribution or the exact chromosomal location of the chosen SNP. The tooltips of the patient displayed in the bottom row provide information about the family and individual identifier as well as the patient's affection status. Further interactions include scrolling, zooming and interactive selection of SNPs of interest, which can then be used for example to be displayed in Mayday's genome browser.

### 3.3 eQTL expression viewer

Using Mayday's strong visualization capabilities, we can visualize the gene expression included in the eQTL dataset in several ways. Here, we use the (enhanced) heatmap view (Gehlenborg *et al.*, 2005), which we find most useful in this context. In this case, the heatmap is enhanced by an additional column showing false discovery rate (FDR)-corrected *P*-values of a *t*-test between ‘affected’ and ‘unaffected’ patients. Other visualizations (profile or parallel coordinate plots, scatter plots, box plots, PCA plots, etc.) can be opened with the click of a button as linked views to further delve into the data.

## 4 APPLICATION TO EQTL DATA

In order to use Reveal for the analysis, several different tab-separated files are required containing the following information: SNP information and gene expression values for each patient, the reference nucleotide for each SNP as well as single- and two-locus results from a PLINK linkage analysis.

To illustrate how Reveal can be used to analyze eQTL data, we applied it to the data provided for the BioVis 2011 Contest (http://it.inf.uni-tuebingen.de/software/reveal). The data encompasses human genomic polymorphisms (SNPs) for 7555 genomic loci, gene expression levels for 15 genes and an associated disease state (‘affected’/‘unaffected’) for a hypothetical spiked-in disease. Sequence data are available for a total of 500 patients of which 193 are affected. Together with the data, results from a statistical analysis using PLINK were also published.

**Fig. 3.**
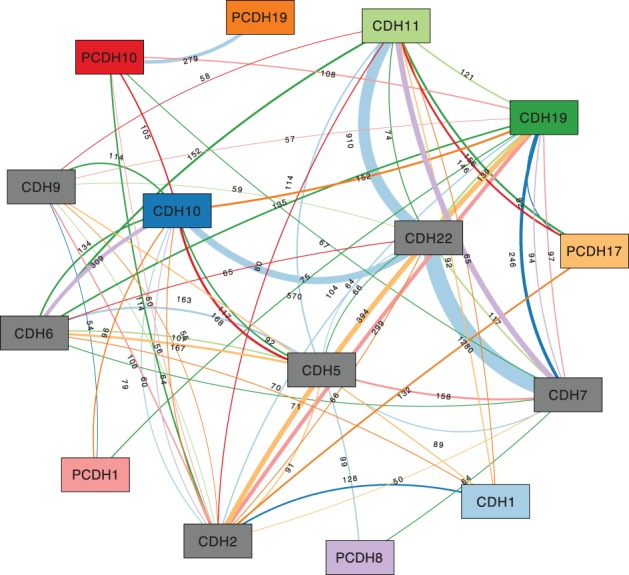
Association graph based on all pairs of 3843 SNPs with a significant association (*P*< 0.05, PLINK two-locus results) with the gene expression of one of 15 genes, showing all edges with weight ≥50. See Figure 1 for a description of the graph's elements

We start our analysis using the results of the two-locus PLINK analysis, which computed statistical significance *P*-values for all pairs of SNPs based on how strongly their presence resp. absence is correlated with the expression of one of the 15 genes. We visualized the results of these 15 PLINK analyses as one association network, covering 62 136 SNP pairs. We chose a minimum edge weight threshold of *τ* = 50 to focus only on very prominent features (Fig. 3). The network impressively visualizes that there are four ‘heavy’ edges with weights ≥400 involving 4 of the 15 genes: CDH22–CDH7, CDH22–CDH11, CDH22–CDH10 and CDH11–CDH7. Of the four genes, two (CDH22 and CDH11) are represented by gray nodes, meaning that there are no SNP pairs associated with their expression. Three of these heavy edges result from SNP pairs significantly associated with the expression of CDH1 and the fourth edge results from SNP pairs significantly associated with PCDH8. Thus, the most relevant SNP pairs in our data show *trans* effects, i.e. they are significantly associated with the expression of a gene at another genomic locus. Furthermore, 7 of the 15 nodes have a high node degree (CDH2, CDH6, CDH7, CDH10, CDH11, CDH19 and CDH22). Interestingly, all nodes sharing a heavy edge also show a high node degree.

Besides two-locus (SNP pair) results from PLINK, Reveal can also import *P*-values computed for the association between single SNPs and gene expression. Including this data in the association graph built from two-locus PLINK data, results in additional filtering possibilities. We chose thresholds to keep only SNPs which have a PLINK regression value (*R*^2^) > 0.1 and a single-locus *P*-value <0.05 in order to concentrate only on very significant associations. 845 SNPs pass this filter based on the single-locus data.

**Fig. 4.**
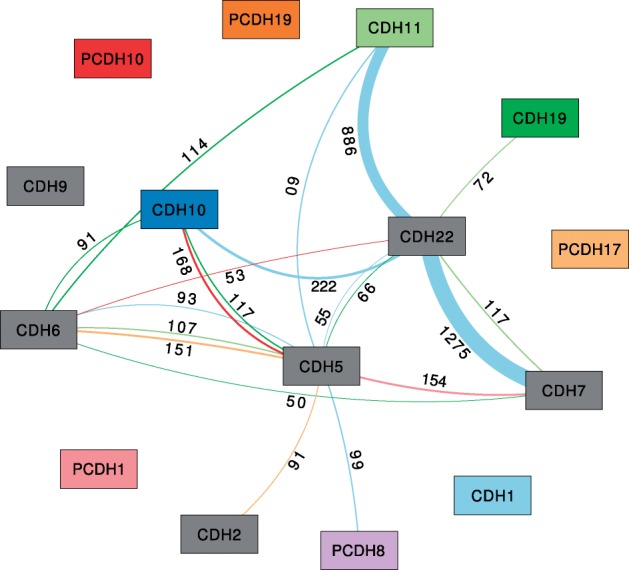
Filtered association graph. Based on the graph shown in Figure 3, only SNP pairs containing at least one highly significant SNP (*R*^2^ > 0.1 and *P* < 0.05, PLINK single-locus result) were retained encompassing a total of 696 different SNPs. All edges with weight ≥50 are shown

Furthermore, we restricted the association graph such that only SNP pairs are kept where at least one SNP partner is among these 845 highly significant SNPs. This left us with 696 SNPs. This smaller graph (shown in Fig. 4) is based on these 696 SNPs. Interestingly, the heavy edges remain almost equally heavy. On the other hand, the degrees of the nodes of CDH2, CDH7 and CDH19, which were the highest in the initial graph, are now greatly reduced, while the node of CDH5 now has the highest degree.

**Fig. 5.**
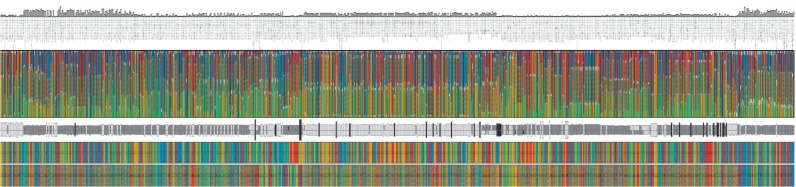
Genotype view showing the 696 SNPs contained in Figure 4. The SNPs were selected such that each of them is part of a highly significant SNP pair, which in addition contains at least one SNP that is statistically significant by itself. In the cohort genotype summary, the boxes for the affected and unaffected group are placed on top of each other which allows to quickly spot interesting columns

In the next analysis step, we want to explore the relevant SNPs of the association network in connection with their genotype distribution within the patient cohort. In particular, we want to find out whether these SNPs (or at least some of them) have different genotypes between the ‘affected’ and ‘unaffected’ patient group. For this, we selected all 696 SNPs involved in the network of Figure 4 and explored them in more detail in the genotype view. This view shows individual patient and reference genotypes, the distribution of the patient cohort genotypes aggregated by meta information (e.g. affected versus unaffected), as well as the distribution of cohort genotypes and the (PLINK) *P*-values associated with each SNP. An overview of all 696 SNPs is provided in Figure 5.

**Fig. 6.**
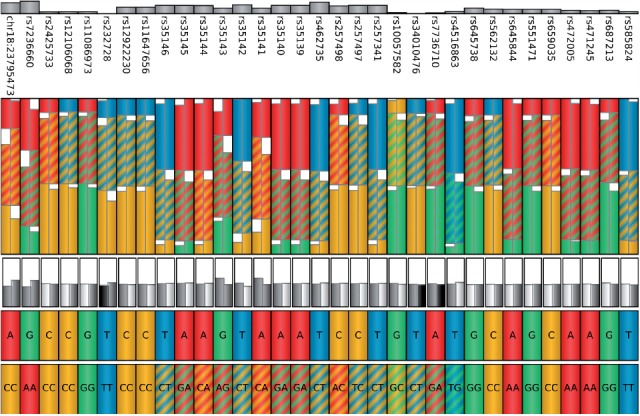
Genotype view showing the 33 SNPs which either have a difference in the simplified cohort phenotype or in consensus strength (by at least 10% points) between the ‘affected’ and the ‘unaffected’ groups

We concentrated on those SNPs that show either a difference in the simplified cohort phenotype (i.e. two different levels of gray in the cohort genotype summary) or a difference in consensus strength (i.e. a difference of at least 10% points between the ‘affected’ and the ‘unaffected’ groups). We found 33 relevant SNPs matching these criteria (Fig. 6). Interestingly, while the differences in cohort group phenotypes are readily apparent from the genotype summary view, the distributions are not very different between the ‘affected’ and the ‘unaffected’group, highlighting the usefulness of summarization based on the simplified genotype.

We also conducted a standard test of independence using Pearson's *χ*^2^ test. The null hypothesis was rejected for 375 of the 696 SNPs (*P* < 0.05). Only 13 of the 375 SNPs are also among the 33 visually identified SNPs. For the other SNPs detected only by the statistical test, the majority of the patients in both groups have the same allele combinations. This shows that visual inspection helped to identify more putatively relevant SNPs than a mere statistical analysis.

**Fig. 7.**
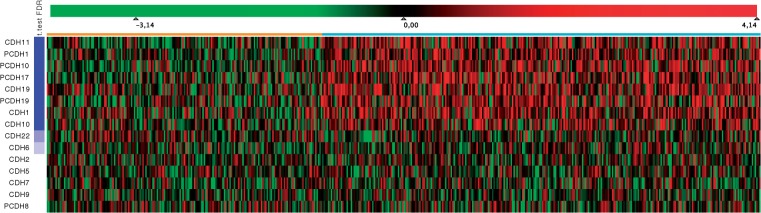
Full heatmap showing expression data for 15 genes in 500 patients. Centered expression values are mapped to a green–red color gradient. Patients (columns) are grouped according to their disease state, indicated by a color bar above the heatmap (affected, orange; unaffected, cyan). Genes (rows) are sorted according to the FDR-corrected *P*-value of a *t*-test (visualized on the left by a blue–white gradient centered on *P* = 0.05) between the affected and unaffected patients' expression values, with the most significant gene on top

**Fig. 8.**
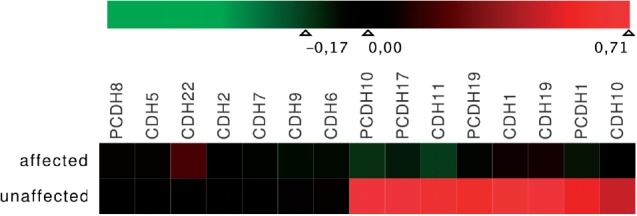
Aggregated Heatmap (transposed). All 500 patients were aggregated into two mean expression profiles according to the patients' disease state (‘affected’ versus ‘unaffected’). Mean expression values were mapped to a symmetrical green–red color gradient centered on zero. Genes (columns) were sorted according to an expression-based clustering (Neighbor joining, Euclidean distance)

Subsequently, we used Mayday to study the gene expression of the 15 genes in the dataset. We conducted a *t*-test between the affected and the unaffected patients' expression values and used FDR-corrected *P*-values to rank the genes according to their significance. The heatmap view (shown in Fig. 7) nicely shows that the genes CDH1, CDH10, CDH11, CDH19, PCDH1, PCDH10, PCDH17 and PCDH19 are differentially expressed between the two groups, which becomes even clearer when aggregating patients according to their disease state (Fig. 8).

Together, heatmap and association graph strongly support the conclusion that the expression of the significantly differentially expressed genes is influenced by SNP pairs forming the heaviest edges in the association graph (i.e. those genes which are indicated by the edge color of the heaviest edges).

**Fig. 9.**
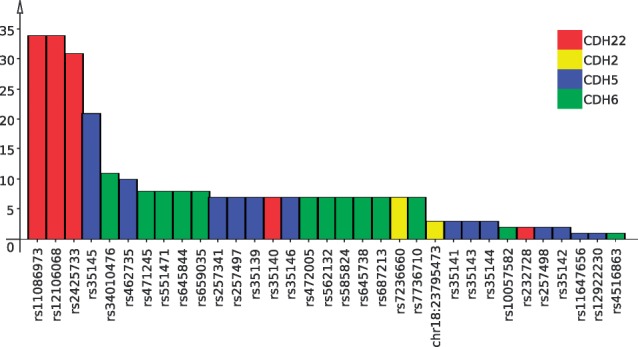
Bar plot showing the 33 SNPs from Figure 6. Each bar shows the number of significant SNP pairs that contain the corresponding SNP

Further analysis of the 33 relevant SNPs showed that these are inside or close to 4 of the 15 genes, namely CDH22, CDH2, CDH5 and CDH6. Notably, these four genes also showed the largest degree in the association graph in Figure 4 after filtering both for significant two-locus and single-locus SNPs. The three SNPs from the CDH22, in addition, are involved in a larger number of significant SNP pairs than the other 30 SNPs (Fig. 9). Interestingly, these 33 SNPs are also only contained in SNP pairs that in combination are associated with genes differentially expressed between ‘affected’ and ‘unaffected’ patients (Fig. 8). We conclude that they are very likely to be correlated with, or even causal for, the disease.

## 5 DISCUSSION

Our visual analytics approach Reveal introduces two different visualizations that allow biologists and bioinformaticians to visually analyze eQTL data. The association graph visualizes significant associations between pairs of SNPs and gene expression. Using the edge color to denote a SNP pair's target gene has the benefit of allowing us to visualize *trans* effects, i.e. SNPs associated with the expression of genes far away from their own genomic locations. At the same time, *cis* effects are also easily visible as edges which are painted in the same color as one of their respective end nodes.

The example data we used here resulted in a relatively small association graph with 15 nodes and a few dozen edges. Larger datasets will result in larger graphs. Our implementation can easily display larger numbers of nodes as well as larger numbers of significant SNP pair associations. However, there is a limit to the complexity of a graph that can be meaningfully interpreted by a human user. Using the filtering based on edge weights, such overly complex graphs can be reduced to focus on the really important associations which are most promising as targets for further research. Although Reveal can handle hundreds or thousands of nodes, it becomes difficult to distinguish the different colors associated with each node, since only about 10 different colors are distinguishable when showing categorial data (Ware, 2004). Furthermore, node-link graphs quickly turn into ‘hairballs’ for large numbers of nodes. Therefore, we introduce interactive highlighting of nodes and edges with identical color in order to maintain usability for large graphs. In addition to the association network, we plan to add a matrix-based view, which does not suffer from the drawbacks of node-link visualizations.

In this context, one can study for example the question, whether the gene whose expression is significantly changed by the respective SNP, is differentially expressed between the different phenotypes. To answer this question, the user can select the respective node within the graph, and a linked heatmap will show the expression of the gene.

The complementary graph of the association network is a SNP graph where SNPs are represented by nodes and an edge is drawn between any two SNPs if they form a significant SNP pair. We plan to combine these two graphs such that for example the user can select edges in the association network and interactively construct a new complementary SNP graph. It would be interesting to study whether there are hubs or cliques in this complementary graph that could indicate whether a specific SNP or SNP pair is relevant for the phenotype.

The connection of the association graph with the genotype view enhances the analytical process since interesting edges can be selected in the association graph and a new genotype view can be generated, visualizing the SNPs associated with these edges. While the information on the cohort genotype distribution is useful for very detailed analyses, our simplified cohort genotype summary view allows for a quick overview of a large number of SNPs. Furthermore, as the genotype distribution of a relevant SNP might only be marginally different between cohort groups (such as the ‘affected’ and ‘unaffected’ groups in our example), the summary can help to highlight cases where the differences are too small to be readily apparent from the full distribution view. This allows users to rapidly narrow their analysis down to interesting SNPs. In combination with filtering techniques, such as presented above, the relevant information can quickly be uncovered even in large datasets. Filtering by *P*-value alone is not always the direct route to meaningful results. To go one step further, we added the possibility to compare individuals to the whole cohort distribution in our genotype view. This enables the user to make predictions on a person's disease state based on the set of selected SNPs of interest. In the current version of the genotype view, it is only possible to distinguish between two states of a phenotype. We plan to enhance this in the future to be able to visualize more disease categories.

To capture the full picture presented by the data, the analysis of expression levels of associated genes is indispensable. Reveal is able to cover all these different aspects of the data through a tight integration into the feature rich expression data analysis framework Mayday.

## 6 CONCLUSION

We present Reveal, an interactive visual tool for the analysis of eQTL data from GWASs. Reveal allows for an efficient analysis of disease-related polymorphisms through a series of different linked visualizations and enables one to study the influences of these polymorphisms on the genotypic as well as on the gene expression level. The two presented views build the basis of the analysis: the association graph summarizes the given PLINK data by generating a network of associations that can be filtered interactively to concentrate on the most important aspects. In the genotype view, SNPs of interest can be visualized in more detail, e.g. to evaluate data from a single patient in the context of the complete cohort of the samples. Being integrated into our visual analytics software Mayday, Reveal can be used for combined analyses of genotypic data, expression data and additional meta-data (e.g. disease phenotype). This makes it a powerful visual analytical tool that combines visualization and interpretation of genome-wide association data with gene expression analysis.

## References

[B1] Agilent (2012). Genespring.

[B2] Battke F. (2010). Mayday—integrative analytics for expression data. BMC Bioinformatics.

[B3] Curtis R. (2011). GenAMap: visualization strategies for structured association mapping. Proceeding BioVis Symposium.

[B4] Dubois P. (2010). Multiple common variants for celiac disease influencing immune gene expression. Nat. Genet..

[B5] Ge D. (2008). WGAViewer: software for genomic annotation of whole genome association studies. Genome Res..

[B6] Gehlenborg N. (2005). A Framework for visualization of microarray data and integrated meta information. Inf. Vis..

[B7] Gibbs R. (2003). The international HapMap project. Nature.

[B8] Golden Helix (2012). SNP and Variation Suite.

[B9] Heinrich J. (2012). iHAT: interactive hierarchical aggregation table for genetic association data. BMC Bioinformatics.

[B10] Holm K. (2010). SNPexp-A web tool for calculating and visualizing correlation between HapMap genotypes and gene expression levels. BMC Bioinformatics.

[B11] Illumina. (2012). Illumina GenomeStudio™.

[B12] Martin O. (2009). AssociationViewer: a scalable and integrated software tool for visualization of large-scale variation data in genomic context. Bioinformatics.

[B13] Mueller M. (2006). eQTL Explorer: integrated mining of combined genetic linkage and expression experiments. Bioinformatics.

[B14] O'Madadhain J. (2005). Analysis and visualization of network data using JUNG. J. Stat. Softw..

[B15] Purcell S. (2007). PLINK: a tool set for whole-genome association and population-based linkage analyses. Am. J. Hum. Genet..

[B16] Ware C. (2004). Information Visualization—Perception for Design.

[B17] Yang T. (2010). Genevar: a database and java application for the analysis and visualization of SNP-gene associations in eQTL studies. Bioinformatics.

[B18] Zou W. (2007). eQTL viewer: visualizing how sequence variation affects genome-wide transcription. BMC Bioinformatics.

